# Factors promoting shared decision-making in renal replacement therapy for patients with end-stage kidney disease: systematic review and qualitative meta-synthesis

**DOI:** 10.1007/s11255-021-02913-8

**Published:** 2021-06-22

**Authors:** Yu Shi, Wang Li, Fangjian Duan, Shi Pu, Hongmei Peng, Mei Ha, Yu Luo

**Affiliations:** 1 School of Nursing, Third Military Medical University (Army Medical University), No. 30 Gaotanyan Street, Shapingba District, Chongqing, 400038 People’s Republic of China; 2 Department of Nephrology, The Key Laboratory for the Prevention and Treatment of Chronic Kidney Disease of Chongqing, Chongqing Clinical Research Center of Kidney and Urology Diseases, Xinqiao Hospital, Army Medical University (Third Military Medical University), Chongqing, 400037 People’s Republic of China

**Keywords:** ESKD, Renal replacement therapy, Decision-making, Qualitative evidence synthesis, Systematic review

## Abstract

**Purpose:**

Shared decision-making (SDM) about the type of renal replacement therapy to use is a matter of great importance involving patients, their families, and health treatment teams. This review aims to synthesize the volume of qualitative work explaining the factors influencing SDM regarding renal replacement therapy.

**Methods:**

A systematic review and qualitative meta-synthesis approach recommended by JBI was used, six databases were searched. Studies were qualitative or mixed research published since 2000, with a primary focus on patient experiences, perceptions and practices regarding which method to choose for renal replacement therapy in End-Stage Kidney Disease (ESKD) patients. All themes were analyzed and compared to the established connectedness.

**Results:**

A total of 1313 patients were enrolled in 32 studies focusing on factors associated with SDM regarding renal replacement therapy were included. All quality evaluations of the literature were medium to high. Four common themes were identified in our synthesis: (1) patient personal reasons, (2) family-related factors, (3) health care professional-related factors, and (4) social factors influence.

**Conclusion:**

The model proposes pathways that could be explored further in future qualitative and quantitative studies and suggests that patients’ beliefs, emotions, and awareness should be targeted alongside patients’ decision-making practices to increase the efficacy of interventions. The majority of studies included in this review focus on older patients, and all report patients’ perspectives. Further research is required to understand the family member perspectives on SMD of renal replacement therapy.

**Supplementary Information:**

The online version contains supplementary material available at 10.1007/s11255-021-02913-8.

## Background

Chronic kidney disease (CKD) has become a global public health problem with occurrence steadily increasing by approximately 6% annually, the global all-age mortality rate from CKD increased 41·5% between 1990 and 2017 [1, 2]. End-stage kidney disease (ESKD) treated by renal replacement therapy: dialysis and kidney transplantation increasing by 43.1% and 34.4% [3].Generally, patients with ESKD represent approximately 0.03% of the total population in many high-income countries, but their treatment alone accounts for approximately 2–3% of the annual healthcare budget [4]. After entering ESKD, patients are faced with a choice of three alternative treatment schemes, including hemodialysis, peritoneal dialysis or kidney transplantation, and it is not easy to identify the most appropriate treatment scheme in the face of different settings. Clinical guidelines point out that advanced CKD patients who need renal replacement therapy should be offered different dialysis modalities and be provided different dialysis options, timely education, and support them to choose the most needed and value-oriented methods [5]. (e.g., hemodialysis vs peritoneal dialysis), there are a number of other decisions made throughout the CKD trajectory, including those related to lifestyle and diet, medication, and advance care planning [6].

Therefore, it is necessary to invoke a doctor-patient shared decision to make a choice. Shared decision-making (SDM) interventions broaden patients' knowledge sources and decrease doctor–patient conflict, promoting decision-making about care and treatment based on informed preferences [7]. SDM means that patients' options are well informed to them and available information could be well considered by them, in that case, decisions are customized to the values and preferences of each patient. Despite SDM emerging as a pillar of national and international quality standards and policies, evidence showed that CKD patients have limited participate in SDM [8]. Regarding SDM, several aspects might jeopardize information transfer [9–12]. First, the accurate information on the expected outcomes of different options may not simply be available or just do not available to some specific group where the patient belongs (generalizability, external validity). Second, such information can be transferred in a biased, non-neutral way. Third, the information is often transferred in a way that could confuse the patient. Fourth, information on outcomes that matters to the patient may not be available, whereas ample information providing on outcomes does not matter to the patient.

Patient decision-making in the management of ESKD has reached increasing attentions. Doctor–patient SDM on the choice of renal replacement therapy is not only reflected in the patient's decision factors but also includes family factors, social factors, and very critical health care factors because much of the decision-making information comes from professional health care teams. There have relevant studies on the integration of viewpoints when patients with ESKD enter the choice of renal replacement therapy [13, 14], including (1) patient's personal reasons, (2) family-related factors, (3) health care professionals-related factors, and (4) social factors. And experiments in the field of SDM on dialysis modality highlight that patients tended to be strongly influenced by the stories of other patients and had far less by the same information provided by a physician [15]. Therefore, the purpose of this review is to examine the patterns and themes of modality decision-making and synthesize these findings into more generalized knowledge claims regarding by meta-ethnography, so as to find out which factors affect the decision-making of ESKD patients mostly and provide an evidence-based foundation for the formulation of doctor–patient SDM assistance schemes in CKD management.

## Methods

### Research design

A meta-synthesis approach (following JBI guidelines [16, 17]) was used to synthesize the qualitative literature on patient decision-making regarding renal replacement therapy. This approach is grounded in pragmatism and phenomenology to assist synthesis of qualitative studies [18]. Meta-synthesis following JBI guidelines [16] is detailed below (and in SI-1).This research work was carried out between March 2020 and January 2021.

### Search strategy

Search using all identified keywords and index terms was undertaken across 9 Chinese and English databases, including PubMed, CINAHL, Embase, Cochrane Library, CNKI, Sinomed, Wanfangand VIP. Collected literatures on decision-making in patients with ESKD from January 2000 to January 2021 (Although research on SDM in patients with ESKD began to appear in 1996, we considered the research at that time to be too old), and the references included in the study were screened. Combination of subject words and free words, and searched all synonyms as far as possible, the search words include End-Stage Kidney Disease/Renal Kidney Replacement Therapy/Shared decision-making/Qualitative research, etc. (in SI-2).

### Inclusion criteria

#### Types of participants

This review considered studies focusing on views and experiences of patients or family members or healthcare professionals (e.g. physicians and/or nurses and/or pharmacists) in decision-making about the treatment of ESKD (defined as using qualitative techniques for recruitment strategies, data collection, and data analysis) (in SI-3).

#### Phenomena of interest

The phenomena of interest for this review were real experience, perceptions and feeling of ESKD patients or family members or healthcare professionals in the SDM of renal replacement therapy.

#### Context

Diagnosis of ESKD, treatment decision RRT and the whole process after selection. These deployment settings were in any country, cultural context or geographical location.

#### Types of studies

Different types of qualitative research, including designs based on phenomenology, ethnography, grounded theory, action research, field research, etc.

#### Assessment of methodological quality

Qualitative papers selected for retrieval were assessed by two independent reviewers for methodological validity prior to inclusion in the review using JBI Qualitative Critical Appraisal Checklist [16] (reported in Table 1). The evaluation contents were 10 items, and each item was evaluated as "Yes", "No", "Not clear" and "Not applicable". The research quality was divided into A, B and C, 3 grades. Quality levels that meet all criteria are A, those that partially meet B, and those that do not meet C at all. All disagreements between the reviewers were resolved after discussing with a third reviewer.

**Table 1:** Characteristics of original qualitative studies synthesized in this review

Study	Country	No. of participants	Aims/objectives	Data collection	Data analysis	Quality appraisals	Summary of key findings
Study 1 [17]	US	14	To explore the considerations taken into account by these patients in decision-making regarding renal replacement therapy	In-depth interviews	Thematic analysis	High	Patients' decisions to decline or accept dialysis are not based on the efficacy of the treatment but rather on personal values, beliefs and feelings toward life, suffering and death, as well as the expected difficulties in fitting the treatment into their life
Study 2 [18]	US	16	To explore how individuals make decisions of 5 chronic kidney disease around vascular access treatment	Mixed method	Thematic analysis	Moderate	Qualitative findings revealed that patient decisions about access were impacted by observations, experiences, and dialogue in the hemodialysis unit
Study 3 [44]	US	20	To figure out how much is attributable to variations in patients with chronic kidney disease, and how a chronic illness context affects choice	Interviews	Thematic analysis	High	Most patients did not perceive themselves to be making an active choice. There was a great deal of patient information, and there were more opportunities to encounter positive information about hemodialysis. A more proactive approach is required to enable patients to engage fully with the dialysis treatment options
Study 4 [47]	UK	179	Better understanding of successful coping strategies will inform patients and help health care providers support patients' needs as they navigate these changes together	Semi-structured telephone interviews	Thematic analysis	High	Learning from the lived experience of others could empower patients to more frequently use positive coping strategies, depending on their personal context, as well as the stage of the disease and associated stressors
Study 5 [48]	GH	2	To explore patient decision-making regarding end-stage kidney disease (ESKD) treatment in sub-Saharan Africa	Interviews	Thematic analysis	High	This study illuminates stark cultural and contextual differences that make decision-making on ESKD treatment a daunting experience for the individual with ESKD in Ghana compared to those in high-income countries. Enhancing information provision would promote informed decision-making, particularly during the initial stages of patient decision-making
Study 6 [49]	AUS	17	To describe the decision-making needs from the perspectives of patients with advanced CKD, professionals, and others involved in the decision	Interviews	Analysis method described but not specified	Moderate	The results revealed evolving decisional needs in five staged through the decision-making journey: 1) progress toward acceptance of dialysis, 2) receiving information, 3) taking some time for personal reflection, 4) seeking opinions and support of others, and 5) re-evaluating one's choice
Study 7 [19]	AUS	13	To examine the personal and structural facilitators and barriers for home dialysis decision-making in older adults with chronic kidney disease	Interviews	Thematic analysis	High	The social and contextual factors associated with age influenced home-dialysis decision-making. Adequate social support, functional status and resources enabled home-dialysis selection. Relevance to clinical practice
Study 8 [20]	CA	146	To explore indigenous End-Stage Kidney Disease (ESKD) patient views on transplantation as a treatment option	Interviews	Thematic analysis	High	Indigenous ESKD patients demonstrated an intense interest in transplantation. Patients believe transplantation is the path most likely to support the re-establishment of their 'normal' family life. Most patients had only a rudimentary knowledge of the notion of transplantation but no understanding of eligibility criteria, the transplant procedure or associated risks. Patients and their families experienced multiple communication barriers that, taken together, undermine their engagement in treatment decision-making. Although cultural sensitivities associated with transplantation were described, these did not constrain patients in making choices about their own health. Transplant units and local treatment providers should develop user-friendly, culturally informed and region-specific education programs regarding transplantation for indigenous ESKD patients
Study 9 [8]	AUS	12	To understand the dialysis modality decision-making process through exploration of the pre-dialysis patient experience to better inform the educational process	Semi-structured interviews	Thematic analysis	High	Modality decision-making is a complex process, influenced by the patient's health literacy, willingness to accept information, pre-dialysis lifestyle, support systems, and values. Patient education requires the flexibility to individualize the delivery of a standardized CKD curriculum in partnership with a patient health care team to fulfill the goal of informed, shared decision-making
Study 10 [22]	UK	29	To understand the experiences of older people (> 70 years) when making a decision about renal replacement therapy	Interviews	Thematic analysis	Moderate	Most were satisfied with the amount of information that they received, although some indicated that the quality of the information could be improved, especially with respect to how daily living can be affected by dialysis
Study 11 [23]	US	11	To understand the nature of illness perceptions in people with ESKD	Interviews	Thematic analysis	High	This study has practical implications for informing practitioners about the psychosocial effects of ESKD diagnosis and treatment
Study 12 [24]	US	10	To determine the views and attitudes of patients who are not on the waiting list regarding the process of transplant allocation	Interviews and focus groups	Thematic analysis	High	Patients trust their caregivers and support an efficacy argument when considering scarce resources. Communication should be improved to ensure clarity and understanding of clinical decisions
Study 13 [25]	Iran	19	To explain the factors influencing decision-making about undergoing PD in end stage renal failure patients	Semi-structured and in-depth interview	Thematic analysis	High	Various personal, family-related, psychological, social, and economic factors could affect the decision on the type of dialysis given to patients. Therefore, basic infrastructures, such as social support, education, and even the specialist and positive perspective of the Ministry of Health, are required to choose this therapeutic method
Study 14 [26]	NED	12	To identify the subjective meanings attached to decisions made by people living with chronic kidney disease as they consider their transition to renal replacement therapy	Semi-structured interview	Thematic analysis	High	This study highlights the importance of optimizing person-centered care and raises important issues for the education and management of people with chronic kidney disease in the pre-dialysis stages of the illness
Study15 [27]	US	9	To gain insight into the decision-making process leading to opting out of dialysis and the experience with conservative non-dialytic management from the patients' perspective	Semi-structured interview	Interpretative phenomenological analysis	High	This study highlights the factors driving patient decisions for conservative non-dialytic management over dialysis to allow medical professionals to offer appropriate support to patients throughout their decision-making process and in caring them for the rest of their lives
Study 16 [29]	US	180	To assist patients with this choice by identifying such factors and effectively provide relevant information	Mixed method	Thematic analysis	High	Incorporation of patient priorities in care improves health outcomes. Given the perceived limited role in the choice of dialysis treatment, our findings support the need for interventions to improve shared decision-making on dialysis treatment options, targeting both patients and clinicians
Study 17 [30]	UK	168	To characterize the experiences of patients beginning RRT	Semi-structured telephone interviews	Thematic analysis	High	Preparing for RRT is an experience rooted in deep feelings of fear. In addition, a number of key factors contributed to patient preparation (or failure to prepare) for RRT. While the education provided by our system was viewed as adequate overall, patients often felt that their emotional and psychosocial needs went unmet, regardless of whether or not they experienced an optimal dialysis start
Study 18 [31]	CA	95	To explore patients’ understanding about choosing between alternative treatments for kidney failure	Interviews	Thematic analysis	High	Patients might choose between therapies based on their perception regarding which therapy most embodies particular characteristics that minimize impact on their lifestyle. Presentation of information regarding RRTs should focus on these characteristics and the potential impact of alternative treatments on the patients and how they wish to lead their lives
Study 19 [35]	CA	9	To gather information about how patients experience involvement in the decision-making process of renal substation therapy just after they have made the decision and before starting dialysis	Interviews	A data-driven analysis based on systematic text condensation Systematic text condensation	Moderate	Patients are a significant part of the decision. Health care professionals contribute to the experience of being involved. Patients delay making the final choice
Study 20 [33]	US	79	To describe the sociocultural factors influencing patients' decisions to remain on dialysis compared to those who sought a transplant	Interviews	Thematic analysis	High	This study identified sociocultural and ethnomedical beliefs and values about the body and transplantation that inform patient treatment decisions. The results emphasize the need for policy makers to recognize patients' decisions when accounting for alleged difficulties in gaining access to transplantation
Study 21 [35]	UK	15	To explore patient perspectives of transitioning from a home-based to an in-center modality	Semistructured interview	Charmaz' constructivist approach	High	Care teams need to offer opportunities to elicit patient knowledge and fears, dispel myths, forge connections with other patients, and visit the dialysis unit before transition to alleviate anxiety. Interventions that facilitate a sense of control should be grounded in the meaning that the disorder has for the person and how it impacts their sense of self
Study 22 [34]	US	10	To explore the experience of the dialysis modality decision-making process from the perspective of a significant other	Semistructured interview	Thematic analysis	Moderate	Significant others play supportive roles for dialysis patients and are involved in the decision-making process associated with treatment decisions. Significant others may have concurrent emotional, informational, and physical needs that affect their role in making and/or implementing the decision
Study 23 [36]	Singapore	23	To explore perspectives on decision-making among older (> / = 70) Singaporean ESRD patients and their caregivers to undergo (or not to undergo) dialysis	Semistructured interview	Thematic analysis	High	While some patients believed that they had made an independent treatment decision, others reported feeling like they had no choice in the matter or that they were strongly persuaded by their doctors and/or family members to undergo dialysis. Patients reported decision-making factors, including loss of autonomy in daily life, financial burden (on themselves or on their families), caregiving burden, alternative medicine, symptoms and disease progression. Caregivers also reported concerns about financial and caregiving burden
Study 24 [37]	US	15	To explores perceptions of older adults with ESKD on HD, specifically their decision to initiate HD, preconceptions and expectations of HD, perceived difficulties with HD, and coping strategies	Interviews	Thematic analysis	High	All participants were reluctant to initiate HD but made the decision on advice from their physicians for varying reasons. Trust in physician opinions also played a role for some. Some participants had positive preconceptions of HD, while a few had negative preconceptions or unrealistic expectations. Even though the majority of participants identified several difficulties with being on HD, they also had positive coping strategies, and the majority indicated that they would make the same decision to initiate HD
Study 25 [38]	US	31	To investigate how ESRD patients and their families make decisions and cope with their circumstances and dialysis treatment	Interviews	Thematic analysis	High	These findings offer insights into chaplain roles in the ESRD setting and the issues that they and other palliative care team members can anticipate and address in patient support and decision-making. The results also support recent work to develop methodologies for research on religious and spiritual issues in medical settings
Study 26 [38]	UK	22	To understand African American patients' knowledge of RRT options and how patient, provider and system-factors contribute to knowledge and preferences	Interviews	Thematic analysis	High	African Americans face significant knowledge and access barriers when deciding on their RRT treatment. Even patients with advanced CKD were still in the early stages of RRT selection. Understanding the knowledge gaps and barriers patients face will inform our subsequent intervention to educate and motivate patients to increase CKD self-care and improve communication among patients, their families and their providers about different RRT treatments
Study 27 [40]	US	9	To explore the decision-making processes of pre-dialysis patients to elucidate how these choices were made	Interviews	Thematic analysis	Moderate	Themes relating to the decision-making process emerged: maintaining one's integrity, forced adaptation, utilizing information, and support and experiencing illness. For renal services, there is a need to tailor information provided to pre-dialysis patients and to be cognizant of the contexts in which they live and operate
Study 28 [41]	US	68	To explore the types of information African American and non-African American patients with chronic kidney disease (CKD) and their families need to inform renal replacement therapy (RRT) decisions	Interviews	Thematic analysis	High	Educational resources addressing the influence of RRT selection on patient morbidity and mortality, autonomy, treatment delivery, and symptoms could help patients and their families select RRT options closely aligned with their values. Including information about the influence of RRT selection on patient personal relationships and finances could enhance resource cultural relevance for African Americans
Study 29 [42]	US	16	To determine patient-perceived factors that influence ESRD patients' choice of dialysis modality among older ESRD patients who are deemed eligible for both PD and HD	Semistructured interview	Thematic analysis	High	Among older ESRD patients who are deemed eligible for both PD and HD, factors relevant to their modality decision-making were identified with respect to physical strength/dexterity and having a sound mind (capability), external forces and constraints (opportunity), and values and beliefs (motivation). Often a combination of factors led to an individual's choice of a particular dialysis modality. However, preferences for PD were primarily based on convenience and maintaining a normal life, while a heightened sense of security was the primary reason for those who selected HD
Study 30 [45]	NZ	17	To explore the link of decision-making by older populations with treatment experiences, implications of dialysis treatment and treatment modality on quality of life, and expectations of aging	Interviews	Iterative analysis	High	Older patients often delay dialysis as an act of self-efficacy. They often do not commit to a dialysis decision following pre-dialysis education. Delaying decision-making and initiating dialysis were common. This was not seen by participants as a final decision about therapy. Predialysis care and education should be different for older patients, who will delay decision-making until the time of facing obvious uremic symptoms, threatening blood tests or paternalistic guidance from their nephrologist
Study 31 [46]	US	14	To explored patients' reasons for choosing conservative management	Semistructured interview	Thematic analysis	High	The information that patients reported receiving from clinical staff differed between units. Patients from units with a more established conservative management pathway were more aware of conservative management, less often believed that dialysis would guarantee longevity, and more often discussed the future with staff
Study 32 [43]	Denmark	13	To explore how patients remained involved in their treatment and care of their own health following a shared decision-making intervention for dialysis choice	Semistructured interview	Systematic text condensation	High	Following the shared decision-making intervention, patients who chose home-based treatment had become more involved in their treatment and care of their own health. The involvement of relatives and support from healthcare professionals contributed positively to this. In contrast, patients who had chosen hospitalbased treatment were less involved in their treatment

#### Data extraction and synthesis

Literature screening and data extraction were conducted independently by two researchers, and mutual verification was conducted. If there were any disagreement, the third researcher shall make the ruling if it cannot be determined after negotiation between the two parties. Through repeated reading, full understanding and reasonable interpretation of the included original research, we grasped the relationship between different research results, interpreted the meaning of different research results, combined similar themes to form a new category, and summarized the new category into integrated results to obtain a new interpretation of the phenomenon. Full texts were imported to NVivo qualitative data analysis software, QSR International Pty Ltd. Version 12 for Qualitative Research. General details of papers were composed of author, published year, methodology, data analysis, setting of the research, demographics of participants, data analysis and conclusion (Table 1).

## Results

### Study characteristics and assessment of methodological quality

**Fig. 1:**
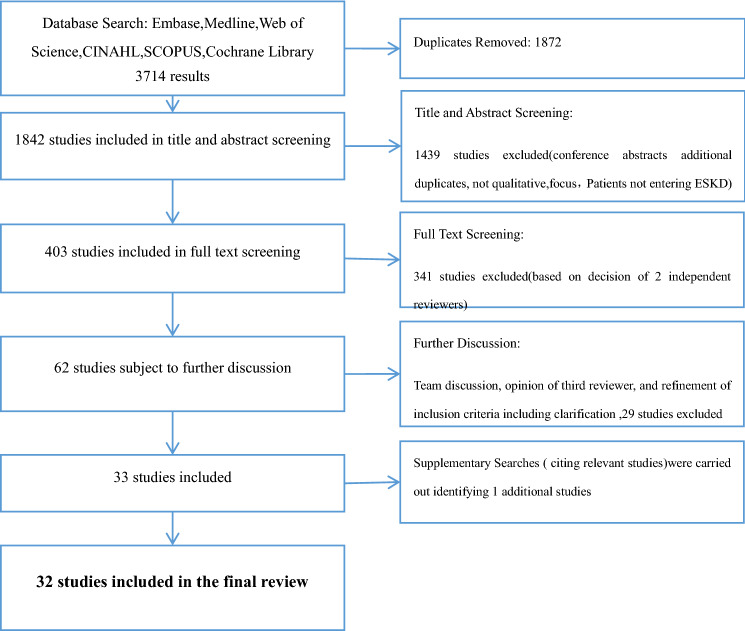
Flow chart illustrating selection of studies through database searches, screening, team discussions and supplementary searches

As shown in Fig. 1, thirty-two studies were included in the final review. The characteristics of these studies are listed in Table 1. Studies represented a total of 1300 ESKD patients (with or without RRT). Two of the included studies (Study 2 & Study 18) were mixed-method studies, and in these cases, only qualitative findings were included.

### Three-dimensional integration of studies (building a whole pattern from the individual parts)

**Fig. 2:**
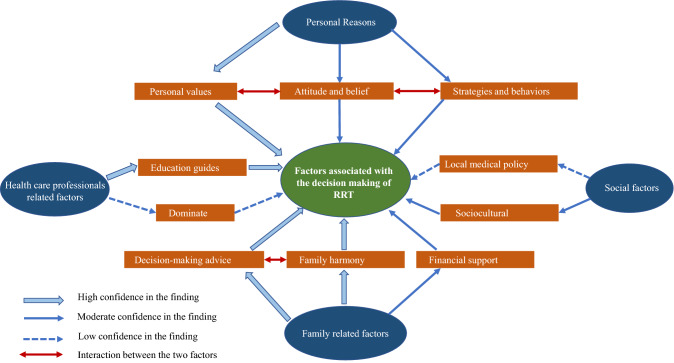
Conceptual model illustrating 4 constructs

By synthesizing the results of these 32 studies, we produced 334 codes. In stage 6 of the analysis, the first reviewer combined similar metaphors to produce 139 s-order constructures. The third constructure is further grouped into four themes [13, 14]: (i.) patients’ personal reasons influence treatment decision-making; (ii.) family-related factors influence patients’ decision-making; (iii.) health care professionals-related factors influence patients’ decision-making; and (iv.) social factors influence patients’ decision-making). The outcome of these themes explains the meaning of these constructures and how they relate to each other, and the final themes and subthemes form a three-dimensional integrated model, as shown in the conceptual model in Fig. 2.

### Theme 1: Patients’ personal reasons influence treatment decision-making

#### Personal values to RRT

It is common to see that before patients make treatment choices, they would explicitly express how they are weighing the benefits and disadvantages of RRT. In other words, will this choice allow them to continue their current lifestyle, or/or may allow them to have more flexibility in their lifestyle? Studies have indicated that a significant group of patients’ value orientation to RRT makes influences to their treatment options.

One of the main reasons why patients refuse dialysis treatment is that they perceive it will deprive them of the right to make their own choices. To them, dialysis will cause more problems, forcing them to give up their freedom and become dependent on medical treatment. In order to be free, they would rather forgo a longer life than to be restricted by a comprehensive treatment such as dialysis [19, 20]. (studies 1, 9, 18).

*“*... *my lifestyle, I am very independent and really didn’t want to be tied to a clinic*.* ”*

*“I am now 77, and you can twist it one way or the other, dialysis is a trouble to go through, it is just a way to postpone death. I don’t see it happening to go to the hospital 3 times per week, I am already too weak, in particular physically. I am worn out!”*

Participants often complained that dialysis treatment wasted their time, not only the treatment itself, the travel to and from the hospital, the waiting and the recovery from the treatment are all added their time costs. They acknowledge that their lives have been changed and it may also continue to have impacts on the future lives [21–24]. (studies 14, 16, 17, 24, 26).

For patients, another key factor in the process of making and implementing decisions is the impact those decisions have on their personal health [25–28]. (studies 7, 15, 21, 22, 32). There are also studies showing that patients' incorrect perception of RRT leads to patient selection [29] (study 5), or patients feel that because of their disease symptoms, they have no choice but to choose dialysis [22, 30–32] (studies 17, 20, 25, 27).

*“I knew that when [I] come to dialysis for, let’s say 10 times or 6 times, it will go. So I was coming here happy since I was going to be OK. ”*

### Strategies and behaviors for dealing with RRT

During the beginning of new activities, the participants initiated some primary changes. This developed from the positive changes in outlook experienced, for example, see previous works [30–34]. Participants would use the time spent on RRTs to do something constructive (studies 4, 6, 17, 20, 25): participate in social activities (such as grocery shopping), get close to nature (*“The doctor told me it was the best thing I could do. Get out and walk.”*), learn a new musical instrument (*“I play guitar for the church and I got back to that”*), perform charity activities (*“I talk to them [other patients with ESKD], sit with them, I explain things to them. In fact, I even support a few of them if they are acutely financially distressed”).*

### Attitude and beliefs to cope with RRT

Patients may also feel fear, denial, regret, anger and shock due to the lack of understanding of their current health condition. Negative emotions, such as fear and ambivalence, often occurred during the preparation period, regardless of whether the patient is in optimal or suboptimal condition at the beginning of RRT. As described by patients, if situation allows, they would like to put off preparatory surgery of dialysis access for as long as possible, and they did not deny that they were willing to delay the dialysis initiation in this way. Numerous factors [22, 31, 32, 34–36] contributed to this sense of fear, including concerns about the AVF placement procedure and/or dialysis, that dialysis may eventually tend to be a death sentence, and lifestyle changes due to the requirements of RRT (studies 5, 6, 12, 14, 15, 25, 27). Despite the difficulties and limitations, most patients are still willing to take whatever steps they can to prolong their lives. *“We all die at some point, and we all know that. Yet, somehow it never dawned on me. It is almost as if I never realized that; I was simply living…. as if life is eternal.…”* Those who chose dialysis showed a desire to survive, and some said they hoped to receive a kidney transplant.. [22, 35] (studies 6, 14). Such optimism made them more positive about the disease and treatment: *“It is not all roses, but with a crisis like this I believe you have, have to stay positive and you have, have to believe in hope.”* The meaning of "being-with" is to provide emotional, physical, psychological and spiritual help/support to the patient. It is also described as general and all-encompassing: *“My part is just to support.”* As another [36]patient shared, “*Being there and listening and supporting the ideas”* (study 17).

### Theme 2: Family-related factors influence patients’ decision-making

#### Giving decision-making advice about RRT

Family is of significance in treatment decision-making, it often plays an indispensable role in persuading patients to start dialysis. In this research, different family-based areas, including high family interactions, good structure, intellectual and cultural background, up-to-date information, and high life expectancy are all very meaningful to the process. On the other hand, inefficiency of family influences and limited knowledge in related fields may play an inhibitory role in this regard. In different studies, families assist patients with kidney disease by supporting and rehabilitating them also influence the selection of their therapeutic method. Some of the families and friends find it very hard to encounter RRT and choose conservative treatment (studies 16, 19, 25).

Studies [31, 33, 34, 37] (studies 3, 4, 5, 20) also show that the support of family members and active participation in the communications of treatment decisions can also help patients to confront the current changes and obstacles.

### (Family) financial factors

When making decisions, the financial burden of dialysis has become the first concern for many patients increasingly, because they perceived those treatment expenses would be extremely burdensome for themselves or even their families. Many studies [21, 33, 34, 36] (studies 4, 5, 15, 17) indicated that it is a huge burden to the patients because they will always need financial support and companion from the family Being financially dependent on their spouse or children for daily and medical needs, the patients may consider it is unnecessary for them to suffer from these additional financial burdens due to the high cost of dialysis.

### Consideration of family harmony

Some patients acknowledged that their family or friends had truly influenced on their decisions of continuing hemodialysis. Some reasons, as cited, are that they wish to see their grandchildren to be grown up or they are caregivers of their spouse or children and are the source of income to the family [22, 26, 30, 38] (studies 4, 6, 25, 31). They discussed several negative impacts they anticipated that dialysis could cause to their lifestyle, yet, for most people, the financial burden of dialysis outweighs the benefits of longer life, even though it may provide the opportunity to spend more time with the family. In some conditions, the patients could also be caregivers to their sick or disabled spouse or children, hence they choose dialysis. In addition, the main reasons for patients to start dialysis could be like, relieving from painful symptoms, making family members happy and satisfied, and prolonging their lives as long as possible., for example, *“I’m doing what [my family] wanted,”* and *“because they wanted me here longer”* [30] (study 25).

### Theme 3: Health care professionals-related factors influence patient decision-making

#### Education guides patients to choose treatment methods

To provide guidance and useful information to patients and prospective dialysis patients en masse, the Renal Nursing Team organized a Renal Patient Information Day. It is the principal channel for these patients to reach information they need. It is generally agreed that this activity plays a crucial or confirmatory role in the participants' subsequent decisions. The team also manages to improve the patient's sensitivity towards their disease. While many patients were satisfied with the care and compassion shown during their contact with team members, some felt there was still room for improvement in their work. The patients emphasized that the team members should give them more empathy and patience, and be willing to answer any questions that patients may have about the preparation and initiation of renal replacement therapy. These studies [22–25, 39–43] (studies 3, 7, 8, 9, 13, 14, 17, 18, 32) shed light on how patients can get the exact treatment. First, patients should have relevant knowledge and educational background to their disease. Patients regard these knowledge and information more as a tool to understand and prepare for dialysis, not just to help them make decisions. Follow-up with doctors and health educators to categorize a wide range of high-quality information, organizing and analyzing relevant information and data are very helpful as well.

### Dominate treatment plan for ESKD patients

The relationship between the patients and their doctors is quite complicated and has impact on their treatment decision-making. These studies [20, 22] (studies 5, 7) indicated that doctors dominate patient decisions. In these studies mentioned above, the relationships between many patients and their doctors may be asymmetrical to some extent, in which the doctor's suggestions and opinions held much more weight than the patients themselves. In that case, the patients felt that they were in no position to consult their doctors and ask suggestions about their disease, but more of educating them than guiding them about medical affairs.

“*There is really nothing to discuss with the doctor. The doctor is wary and persuaded me to accept dialysis… all they would do is to encourage me to go on dialysis and tell me the benefits of dialysis.”*

Some participants have made up their mind to initiate HD because of the physicians’ opinions, and they trusted them. Longer-term doctor–patient relationships and the accompanying higher trust levels typically made patients more comfortable with their decision to begin treatment.

*“And, it was much easier, I think, than if you have some, you know, you’re thinking, ‘Oh, he’s just trying to make money’ or, you know, whatever. But I trusted her [my doctor].”*

### Theme 4: Social factors influence patient decision-making

#### Local medical policy

The only study [29] (study 5) in developing countries indicated that medical policies have an impact on patient treatment choices. For many patients, the location of the treatment center greatly influences (limits) their choice of treatment. In fact, for some people, the only realistic option is to move or to travel long distances across regions to get treatment for renal services. As in many high-income countries, palliative care for ESKD in Ghana is not an option that can be openly considered.

#### Sociocultural

These studies [20, 21, 23, 41] (studies 4, 5, 7, 13) suggest that religious beliefs are significant in helping patients and their families dealing with dialysis. Faith brings hope and fear to the patients and their families. "*I have no worries about it. Not at all. Yah, if you have that [faith], there’s nothing to fear. No fear of death.*" said a man who was anticipating his death after discontinuing dialysis. Another patient who chose to stay on dialysis had a similar response. While some participants found pleasure in the close relationship between their grandchildren, others said faith and religion played a major role in how they coped with a medical diagnosis and received hemodialysis.

## Discussion

In this study, the researchers have reviewed thirty-two latest qualitative studies mainly from websites, professional magazines on SDM about the categories of renal replacement therapy and finally synthesized numbers of findings. Meta-synthesis was included in the studies[44], and secondary analysis of the data are presented in these studies. A conceptual model (Fig. 2) was produced illustrating the relationships among our main themes: (i) patients’ personal reasons; (ii) family-related factors; (iii) health care professionals-related factors; and (iv) social factors. Detailed factors and explanations have been proposed in the current qualitative literature.

In theme 1, the studies indicated that bothers came from various sources with provided evidences, and stress is associated with all sorts of personal reasons, which influences treatment decision-making. Patients cannot refuse to deal with kidney failure and its impacts on their lives and lifestyles over years and years. For example, diet and fluid restrictions, mental burdens and social stresses, such as loss of independence, social stigma, and travel limitations. They all have deep impact on employment and social relationships for CKD patients, in spite of before or after dialysis. To keep the stability of pre-dialysis, life quality of patients is a key to the smooth and successful treatment intervention and patient buy-in; therefore, this is a must-consider item for dialysis modalities. Meanwhile, the transfer of key information and knowledge and patient’s decision-making are influenced by their emotions, including, fear and denial, such emotions came from inexperience and misunderstanding of their disease and what do they actually need during the treatment process, these are all negative influences when making subsequent decisions after receiving relevant information, thus the willingness of patients could be reduced. This happens to hold similar view and agrees with an earlier finding that emotions could interfere patients with information and background knowledge acquisition, and how they consider their dialysis experience. So the patients with fear of unknown consequences may lead them to perceive the choices as being more risky. To make more sensible and relatively correct decisions, screening known situations to identify bad emotions requires the intervention of various social work, and if necessary, the making of major treatment decisions should be postponed.

Based on a plenty of qualitative and quantitative studies and reports, family atmosphere and geographical location play a very important and decisive role in determining the therapeutic methods for ESRD patients. This is especially true in Iran and some Western countries where families have a high level of support for patients.[11, 12]. When choosing PD as therapeutic method, different regions and family atmosphere have both promoting and inhibiting effects on the patient's decision-making. This paper mainly discusses that different family backgrounds, such as good family interaction, structure, education level, ability of up-to-date knowledge acceptance, and expectation of life expectancy, may play a role in promoting the treatment of patients. While lower efficiency, economic ability and knowledge level of family members may inhibit the effect. In different studies, the role of families in the treatment process, in terms of supporting and helping them with getting better from disease, influenced their choice of therapeutic method [9]. In this study, we hope to conduct more investigations and studies on the influencing factors of patients, including the influences from themselves and their families, during the decision-making process of choosing treatment methods. We also believe that a more comprehensive understanding and analysis of these factors can lead to decisions that provide a better quality of life for patients.

In the future, if time and source of the guidance to patients could be tailored to their preferences and focus on their needs, it is possible that under such method, patient is highly expected to be more engaged into decision-making. For patients, adequate time and information can help them learn about which treatments are more appropriate to their own lifestyles, so they can be well prepared than willing to start dialysis. Only one optional curriculum cannot be suitable for all patients, they need more options. So we must come up with treatment plan one on one on the basis of patients’ needs and requests. This means we need to develop a flexible CKD education curriculum and information resources library to tailor treatment models to patients, which requires patients to fully understand risks and benefits, and to deal with modality information in a practical way [45, 46]. We often find that certain clinicians may have a limited ability to communicate risks and outcome probabilities with individualized patients, especially for those who are old. What is more, a clearer and more improved role clarification is also required, as patients frequently fail to identify their roles precisely. Studies [34, 36] have shown that classes should contain caregiver-focused information and coping skills. In general, medical staff should adopt a scientific method of education and guidance and fully respect the patient's right to participate in treatment decision-making.

In addition, sociocultural diversity requires us to take into account the diverse population of ESKD patients who have different ethnic, religious and socioeconomic backgrounds. In countries influenced by traditional Confucian culture, including China, the freedom of individual decision-making is relatively weak, and the family plays an extremely important role in it. Italian culture is dominated by the nuclear family, so patients' decisions are more influenced by the family. Especially in patients with religious beliefs, most of their views on the disease maintain a positive attitude. Elderly patients with religious beliefs tend to choose conservative treatment because of their personal beliefs.

## Conclusion

This review aims to use a meta-synthesis approach [44] to synthesize thirty-two recently published qualitative studies. This research proposed what is worthy of further study and discussion in future qualitative and quantitative research, and suggested that the practice of doctor–patient SDM should be closely combined with the patient's belief, emotion and consciousness so as to improve the efficiency of intervention. Most of the studies used in this review focused on older patients, some of their perspectives have been reflected and presented on this paper. Further research is needed to understand the perspectives of family members on renal replacement therapy decisions.

## Supplementary Information

Below is the link to the electronic supplementary material.

Supplementary file1 (DOCX 14 KB)

Supplementary file2 (DOCX 13 KB)

Supplementary file3 (DOCX 13 KB)

## Data Availability

PubMed, CINAHL, EMBASE, Cochrane Library, and China National Knowledge Infrastructure (CNKI) databases were used to identify all relevant published articles for review. These articles are open to the public.
