# Supplementary material for: Factors promoting shared decision-making in renal replacement therapy for patients with end-stage kidney disease: systematic review and qualitative meta-synthesis
Source: Int Urol Nephrol. 2021 Jun 22;54(3):553–74. doi: 10.1007/s11255-021-02913-8 (PMC8831292; doi:10.1007/s11255-021-02913-8)
Supplement: Supplementary file 1 — Supplementary file1 (DOCX 14 KB) [file 11255_2021_2913_MOESM1_ESM.docx]

**SI-1** Meta-analysis stages, steps, and tools/software used to review and synthesize studies

| Major stages of research | Steps | Tools/Software Used |
| --- | --- | --- |
| 1.Identifying the main research methods | 1.Select a qualitative synthesis approach appropriate for review question | Refers to the standard process of qualitative research of JBI |
| Stage 1: Getting started | 1. Preliminary literature searches | Databases (Embase, Medline) |
|  | 2. Register review protocol | PROSPERO (Registration) |
| Stage 2: Deciding what is relevant to the initial interest | 1.Develop search strategy and run exhaustive search of databases | Databases: Embase, Medline, Web of Science, CINAHL, SCOPUS, Cochrane Library |
|  | 2. Title and abstract screening | EndNote |
|  | 3. Full text screening | EndNote |
|  | 4. Team discussions about discrepancies |  |
|  | 5. Supplementary searches | Reference lists, ‘Cited by’ tools on Google Scholar |
| Stage 3: Reading the studies | 1. Data extraction (full texts) | NVivo |
|  | 2. Noting initial observations | Memos in NVivo |
|  | 3. Extract key contextual information and key findings | NVivo (to organize data) |
|  |  | Microsoft Word (to visualize data in table format) |
| Stage 4: Determining how the studies are related | 1. Consider similarities and differences across studies | Matrix in NVivo |
|  |  | Table in Microsoft Word |
| Stage 5: Translating the studies into one another | 1. Enter key contextual information for each study to preserve context and meaning of original studies throughout the analytic process. | Microsoft Excel spreadsheet |
|  | 2. Enter metaphors (findings from each study) into table (row for each study, column for each new metaphor not already reported by a previous study). If studies reported similar findings under different names or themes, these findings were entered into the same column, and a metaphor name was selected that best represented all of the data. | Microsoft Excel spreadsheet |
|  | 3. Compare each study against all previous studies, observing initial similarities (reciprocal translations) and differences (refutational translations) between studies. | Microsoft Excel spreadsheet |
|  | 4. Color coding 1st order (participant quotes), 2nd order (primary study author) and 3rd order (reviewer) interpretations to preserve context and meaning. | Microsoft Excel spreadsheet |
| Stage 6: Synthesizing translations | 1. Read Excel file row by row summarizing similarities and differences of each study (reciprocal and refutational translations). | Microsoft Excel spreadsheet |
|  | 2. Read Excel file column by column to define, refine and summarize each metaphor while observing similarities and differences across studies. | Microsoft Excel spreadsheet |
|  | 3. Group similar metaphors (original findings) together into 3rd order constructs (categories developed by reviewer). | Microsoft Word |
|  | 4. Develop themes that describe constructs and relationships between them. | Microsoft Word |
|  | 5. Map relationships between key themes within each individual study. | Conceptual models using paper and pen |
|  | 6. Integrate individual conceptual models to form an overarching conceptual model of relationships between constructs across studies. | Conceptual model (Microsoft PowerPoint) |
| Stage 7: Expressing the synthesis | 1. Write a summary of each theme supported by quotes. | Microsoft Word |
|  | 2. Rewrite theme summaries considering confidence and alternative interpretations. | Microsoft Word |
