# Supplementary material for: Factors promoting shared decision-making in renal replacement therapy for patients with end-stage kidney disease: systematic review and qualitative meta-synthesis
Source: Int Urol Nephrol. 2021 Jun 22;54(3):553–74. doi: 10.1007/s11255-021-02913-8 (PMC8831292; doi:10.1007/s11255-021-02913-8)
Supplement: Supplementary file 2 — Supplementary file2 (DOCX 13 KB) [file 11255_2021_2913_MOESM2_ESM.docx]

**SI-2** Search strategy used to identify qualitative studies on decision making about the type of RRT published since 2000

| Search terms (based on key words of relevant articles and test searches in Medline and Embase; terms and search operators varied slightly according to database guidelines) | |
| --- | --- |
| Focus | End (-) Stage Kidney\Renal Disease; Disease, End(-)Stage Kidney\Renal; Chronic Kidney\Renal Failure; Kidney\Renal Failure, Disease; Kidney\Renal) Disease, End (-) Stage; ESRD; ESKD |
|  | Renal\Kidney Replacement Therap(y\ies); Therap(y\ies), Renal\Kidney Replacement; Replacement Therap(y\ies), Renal\Kidney |
|  | Shared decision making; Decision Support Techniques; Decision\Policy Making; Decision Making, Shared |
| Research method | Qualitative; Qualitative research; Qualitative study; Qualitative method; Interview(s/ing/ed); Focus group(s); Phone(s/call);Diary/diaries; Photo(s); Memo(s); Qualitative analysis; Thematic analysis; Content analysis; Grounded theory; Phenomenological analysis; Discourse analysis; Narrative analysis; Observ(e/ed/ing/ation(s) |
| Target participant | End stage renal disease patients |
| Search limits | 2000-2020 |
| Databases (selected to span psychology, social science and medical disciplines | Embase, Medline, Web of Science, CINAHL, SCOPUS, Cochrane Library |
| Date of final database search | 16-Jan-21 |
| Supplementary search strategies | Searching research citing relevant studies |
