# Supplementary material for: Factors promoting shared decision-making in renal replacement therapy for patients with end-stage kidney disease: systematic review and qualitative meta-synthesis
Source: Int Urol Nephrol. 2021 Jun 22;54(3):553–74. doi: 10.1007/s11255-021-02913-8 (PMC8831292; doi:10.1007/s11255-021-02913-8)
Supplement: Supplementary file 3 — Supplementary file3 (DOCX 13 KB) [file 11255_2021_2913_MOESM3_ESM.docx]

**SI-3** Inclusion and exclusion criteria for title, abstract and full text screening

|  | Inclusion Criteria | Exclusion Criteria |
| --- | --- | --- |
| Methodology | Qualitative studies (using both qualitative methods and analysis). Mixed methods studies in which the qualitative component can be extracted | Quantitative studies Review articles Intervention studies (evaluations of interventions) |
| Dates | Published between 2000 and March 2020 | Published before 2000 |
| Language | English,Chinese | Any language other than English and Chinese |
| Target Age | Adult | Children from one year to young adult |
| Focus | Experiences, perceptions and factors about decision making on the type of renal replacement therapy (min. one relevant sentence in abstract during title and abstract screening; author stated relevant aim or objective in full text screening) | Intervention implementation studies on decision making about the type of renal replacement therapy |
| Participants | End stage renal disease patients, | Patient family members, healthcare professionals |
